# Changes in L‐phenylalanine concentration reflect and predict response to anti‐PD‐1 treatment combined with chemotherapy in patients with non‐small cell lung cancer

**DOI:** 10.1002/mco2.70100

**Published:** 2025-02-17

**Authors:** Yaqing Liu, Yu Ping, Liubo Zhang, Qitai Zhao, Yachang Huo, Congcong Li, Jiqi Shan, Yanwen Qi, Liping Wang, Yi Zhang

**Affiliations:** ^1^ Biotherapy Center and Cancer Center The First Affiliated Hospital of Zhengzhou University Zhengzhou Henan China; ^2^ Department of Oncology The First Affiliated Hospital of Zhengzhou University Zhengzhou Henan China; ^3^ State Key Laboratory of Esophageal Cancer Prevention and Treatment Zhengzhou Henan China; ^4^ School of Life Sciences Zhengzhou University Zhengzhou Henan China; ^5^ Tianjian Laboratory of Advanced Biomedical Sciences Academy of Medical Sciences Zhengzhou University Zhengzhou Henan China; ^6^ School of Public Health Zhengzhou University Zhengzhou Henan China

**Keywords:** immunotherapy, non‐targeted metabolomics, NSCLC, serum metabolomics

## Abstract

Chemotherapy combined with checkpoint blockade antibodies targeting programmed cell death protein (PD‐1) has achieved remarkable success in non‐small cell lung cancer. However, few patients benefit from long‐term treatment. Therefore, biomarkers capable of guiding the optimal therapeutic selection and reducing unnecessary toxicity are of pressing importance. In our research, we gathered serial blood samples from two groups of non‐small cell lung cancer patients: 49 patients received a combination of therapies, and 34 patients went under chemotherapy alone. Utilizing non‐targeted metabolomic analysis, we examined different metabolites’ disparity. Among the lot, L‐phenylalanine emerged as a significant prognostic marker in the combination treatment of non‐small cell lung cancer patients, interestingly absent in patients under sole chemotherapy. The reduced ratio of L‐phenylalanine concentration (two‐cycle treatment vs. pre‐treatment) was associated with improved progression‐free survival (hazard ratio = 1.8000, 95% confidence interval: 0.8566‒3.7820, *p* < 0.0001) and overall survival (hazard ratio = 1.583, 95% confidence interval: 0.7416‒3.3800, *p* < 0.005). We further recruited two validation cohorts (cohort 1: 40 patients and cohort 2: 30 patients) to validate the sensitivity and specificity of L‐phenylalanine prediction. Our results demonstrate that a model based on L‐phenylalanine variations could serve as an early risk‐assessment tool for non‐small cell lung cancer patients undergoing treatment, potentially facilitating strategic clinical decision‐making.

## Graphical abstract

Liu et al. studied serum from patients with non‐small cell lung cancer before, during, and after treatment with anti‐programmed cell death protein‐1 plus chemotherapy, and revealed the metabolic heterogeneity of responders and non‐responders. The combination of clinical data profiling and change of L‐phenylalanine concentration collected pre‐ and post‐treatment presented a strong predictor for response to immunotherapy in a minimally invasive manner.

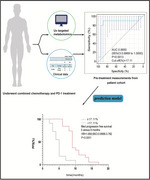

## 1 INTRODUCTION

Non‐small cell lung cancer (NSCLC) is one of three tumors with the highest incidence rates in the world,^1^ and most patients are diagnosed at an advanced stage.^2^ Over the past decade, immunotherapy has shown great potential in the treatment of NSCLC.^3^ Currently, it is used as a first‐line treatment for advanced metastatic non‐small cell lung cancer and as an adjuvant therapy for patients who cannot undergo surgery.^4^

According to the National Comprehensive Cancer Network guidelines, PD‐L1 is the only recommended biomarker for metastatic NSCLC.^5^ However, if a patient is negative for PD‐L1 expression, it is difficult to predict the efficacy of PD‐1/PD‐L1 inhibitors.^6^ Based on our clinical findings, patients who do not express PD‐1/PD‐L1 can benefit from immunotherapy,^7^ whereas some patients with greater than 50% PD‐1/PD‐L1 expression develop resistance to early treatment. Therefore, the use of PD‐1/PD‐L1 expression alone to predict or evaluate treatment response is inaccurate. Other biomarkers currently available for monitoring patient responses to immunotherapy include the tumor mutation burden (TMB),^8^ target gene mutations (KRAS, EGFR, ALK, etc.),^9^ and circulating tumor genes (ctDNA).^10^ However, these invasive tests cannot be repeated to monitor a patient's response to immunotherapy promptly. Therefore, developing clinically useful predictive biomarkers to accurately identify patients who may benefit from treatment can help clinicians adjust treatment strategies on time and provide more treatment opportunities for patients.

Currently, common blood biopsy methods used in clinical and research studies include exosomes (EVs),^11^ tumor‐induced platelets (TEP),^12^ and microbiota and microbial metabolites.^13^, ^14^, ^15^, ^16^ Blood biopsy samples have a short half‐life and require rapid processing with strict requirements for sampling processing time. Currently, standardized analytical methods are lacking. Therefore, our study began from the perspective of metabolomics, which is the closest link to phenotypes in the biological framework of gene‐transcription‐protein metabolism.^17^ Changes in the genome and proteome can be represented by metabolomics as genes and proteins regulate metabolic pathways and rates, thereby affecting the concentration of small molecules in the body.^17^ These metabolites directly reflect the physiological and pathological changes that occur in organisms, and metabolomics is directly correlated with changes in biological phenotypes.^18^ Furthermore, the model could easily help clinicians decide whether to continue PD‐1Ab combined with chemotherapy treatment, sampling in real‐time repeatably, and more cheaply than imaging, and support higher patient compliance.

In our study, a comprehensive analysis was performed to identify the serum metabolites that predict the effectiveness of immunotherapy combined with chemotherapy, and to determine whether changes before and after treatment can be used as prognostic of response to combination therapy in NSCLC. These predictions were validated using independent randomized cohorts. Compared with before treatment, an increase in the concentration of serum metabolic biomarkers is associated with the survival of patients with non‐small cell lung cancer receiving combination therapy.

## 2 RESULTS

### 2.1 Patient cohorts and baseline characteristics overview

**FIGURE 1:**
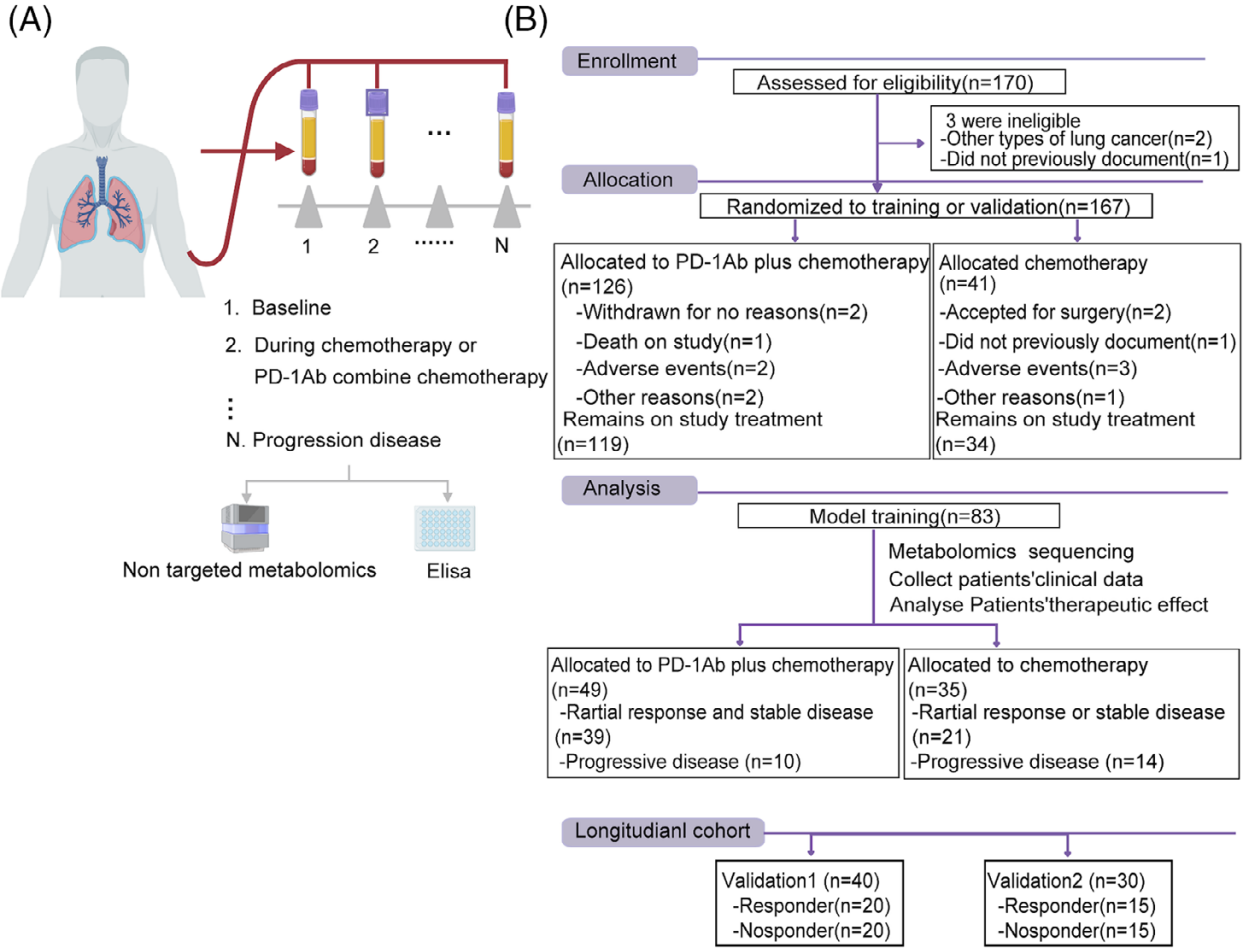
Patients with chemotherapy treatment or chemotherapy plus PD‐1Ab and throughout the study treatment. (A) Treatment schedule outline of the clinical trial of treatment (pemetrexed and platinoid) plus PD‐1Ab or mono‐chemotherapy (pemetrexed and platinoid), and sample collection time point (grey arrow). Serum from peripheral blood at the end of each treatment cycle is used for non‐targeted metabolomics and Elisa kit. (B) Cohort derivation, validation, and longitudinal assessment.

Patients with NSCLC who received chemotherapy alone or chemotherapy combined with anti‐PD‐1 antibodies were enrolled in this study. Peripheral blood was collected from each patient to separate serum and PBMC after every cycle of treatment (Figure 1A). All patients were evaluated for clinical efficacy at baseline and after every treatment cycle, according to RECIST 1.1. Overall, 170 patients with lung cancer were enrolled in this study; 126 NSCLC patients received chemotherapy combined with anti‐PD‐1 antibody and 41 NSCLC patients received chemotherapy alone after screening (Figure 1B). Seven patients were excluded for some reason (death, adverse events, or side effects); remaining patients were divided into three cohorts: discovery cohort (*n* = 83), validation cohort 1 (*n* = 40), and validation cohort 2 (*n* = 30). The baseline clinical characteristics of all the participants (39 responders and 10 non‐responders) are presented in Table 1.

**TABLE 1:** Baseline characteristics of enrolled patients.

		Discovery cohort			Validation cohort 1			Validation cohort 2		
		R	NR	*p*‐value	R	NR	*p*‐value	R	NR	*p*‐value
**Age**	**Average**	59 (44–73)	56 (48–71)	0.3350	59.5 (51–73)	61.5 (46–72)	0.7529	58.87 (51–79)	57.785 (47–75)	0.5178
	**Female**	16	6		5	7		8	6	
**ECOG**	**0**	22	4	0.3536	14	12	0.5073	6	5	0.7048
	**1**	17	6		6	8		9	10	
**Expression of PD‐L1**	**<1%**	14	3	0.6250	6	9	0.0474	4	6	0.0425
	**≥1% and <49%**	9	2		3	6		2	6	
	**≥50%**	11	2		10	2		8	1	
	**Unknown**	5	3		1	3		1	2	
**Surgery**	**Yes**	6	3	0.2869	5	2	0.2119	3	4	0.6660
	**No**	33	7		15	18		12	11	
**Tumor**	**0**	1	0	0.6614	1	0	0.0956	0	0	0.6065
	**1**	5	1		0	1		7	6	
	**2**	16	2		9	7		4	3	
	**3**	10	4		9	5		2	1	
	**4**	7	3		1	7		2	5	
**Node**	**0**	5	0	< 0.05	3	5	0.599	3	1	0.6295
	**1**	3	0		2	1		5	6	
	**2**	7	6		7	4		4	3	
	**3**	24	4		8	10		3	5	
**Metastasis**	**0**	19	4	0.6221	11	6	0.1098	7	4	0.2557
	**1**	20	6		9	14		8	11	
**Disease stage**	**1**	5	0	0.107	2	1	0.8760	3	1	0.1395
	**2**	6	1		2	3		5	1	
	**3**	6	5		5	4		4	7	
	**4**	22	4		11	12		3	6	
**Number of tumor metastasis**	**0**	19	3	0.0736	11	6	0.3104	5	1	0.1942
	**1**	10	1		1	2		4	6	
	**2**	6	1		3	2		3	1	
	**3**	3	4		4	5		2	6	
	**5**	1	1		1	5		1	1	
**Brain metastasis**	**Yes**	5	2	0.5627	4	8	0.1675	2	4	0.3613
	**No**	34	8		16	12		13	11	
**Pleural metastasis**	**Yes**	10	3	0.7806	8	10	0.5250	3	5	0.4090
	**No**	29	7		12	10		12	10	

*Note*: Chi‐square test for responders versus non‐responders.

Abbreviations: NR, non‐responders; R, responders.

### 2.2 Non‐targeted metabolomics screening and analysis for serum differences

**FIGURE 2:**
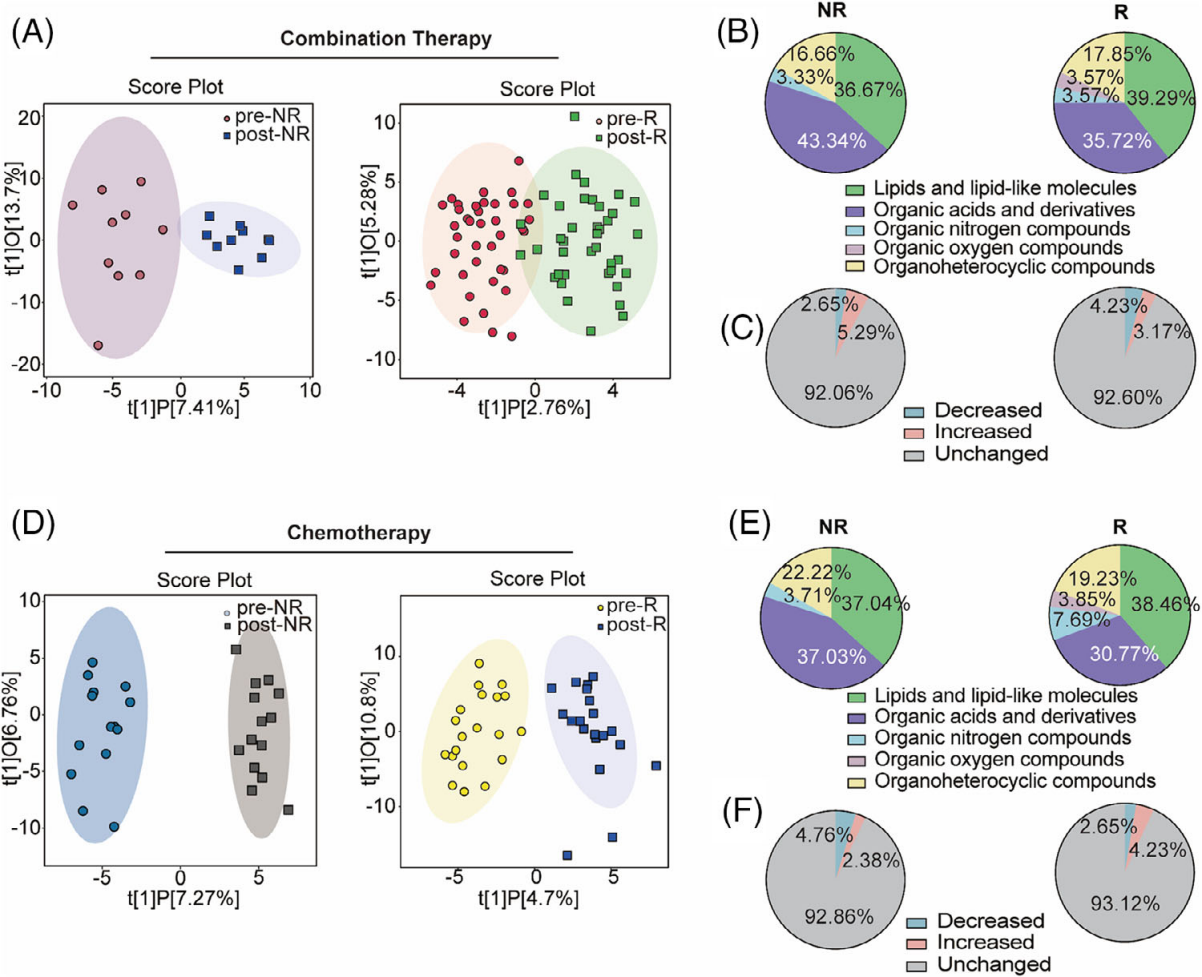
Examination of the metabolic profiles of serum specimens. (A) Scores plot of orthogonal partial least squares discriminant analysis (OPLS‐DA) of samples that received combination therapy in the discovery set based on the paired before and after treatment features. Left: non‐responder (NR) samples before (grey, pink) and after (grey blue) chemotherapy combine PD‐1Ab; right: responder (R) samples before (light pink) and after (green) chemotherapy combined with PD‐1Ab. (B) Components of the changed serum metabolite in patients before and after combination therapy. Left: NR; right: R. (C) Metabolomics analysis of serum metabolites in patients with combination therapy. Left: NR; right: R. (D) Scores plot of OPLS‐DA of samples received chemotherapy alone in the discovery set. Left: NR samples before (blue) and after (grey) chemotherapy. Right: R samples before (yellow) and after (light blue) chemotherapy. (E) Components of the changed serum metabolite in patients before and after treatment with chemotherapy. Left: NR; right: R. (F) Metabolomics analysis of serum metabolites in patients with chemotherapy. Left: NR; right: R.

Serum samples at the initiation of treatment (baseline) and two cycles after treatment completion (post‐treatment) were collected from 83 patients with NSCLC and used for non‐targeted metabolomic analysis. Pooled quality control (QC) samples, correctly clustered based on OPLS‐DA (Figure S1A,B), verified the stability and repeatability of the sample analysis sequence. Orthogonal partial least squares discriminant analysis (OPLS‐DA) revealed that the metabolite features of NSCLC patients treated with chemotherapy combined with anti‐PD‐1 antibodies differed (Figure 2A). In the non‐response (NR) group, different metabolites between the baseline and post‐treatment were enriched in organic acids and their derivatives. In contrast, metabolites in the response (R) group exhibited change in lipids and lipid‐like molecules, organic acids and derivatives, and organ heterocyclic compounds (Figure 2B). Furthermore, the proportion of differential metabolites accounted for 7.94%, of which 5.29% were upregulated and 2.65% were downregulated in the NR group. The proportion of differential metabolites in R group accounted for 7.4%, with 3.17% representing increased metabolites and 4.23% representing decreased in metabolites (Figure 2C). The OPLS‐DA analysis of the NR group treated with chemotherapy alone highlighted a unique metabolic landscape before and after treatment, the visualization clearly separating the NR and R groups, similar to Figure 2A (Figure 2D). In the NR and R groups that received chemotherapy alone, the altered metabolites were mainly concentrated in lipids and lipid‐like molecules, as well as organic acids and derivatives (Figure 2E). In all, 7.14% of metabolites were different between baseline and post‐treatment in the NR group, and 6.88% of metabolites were different in the R group (Figure 2F).

### 2.3 Identification of metabolites predictive of chemotherapeutic response

**FIGURE 3:**
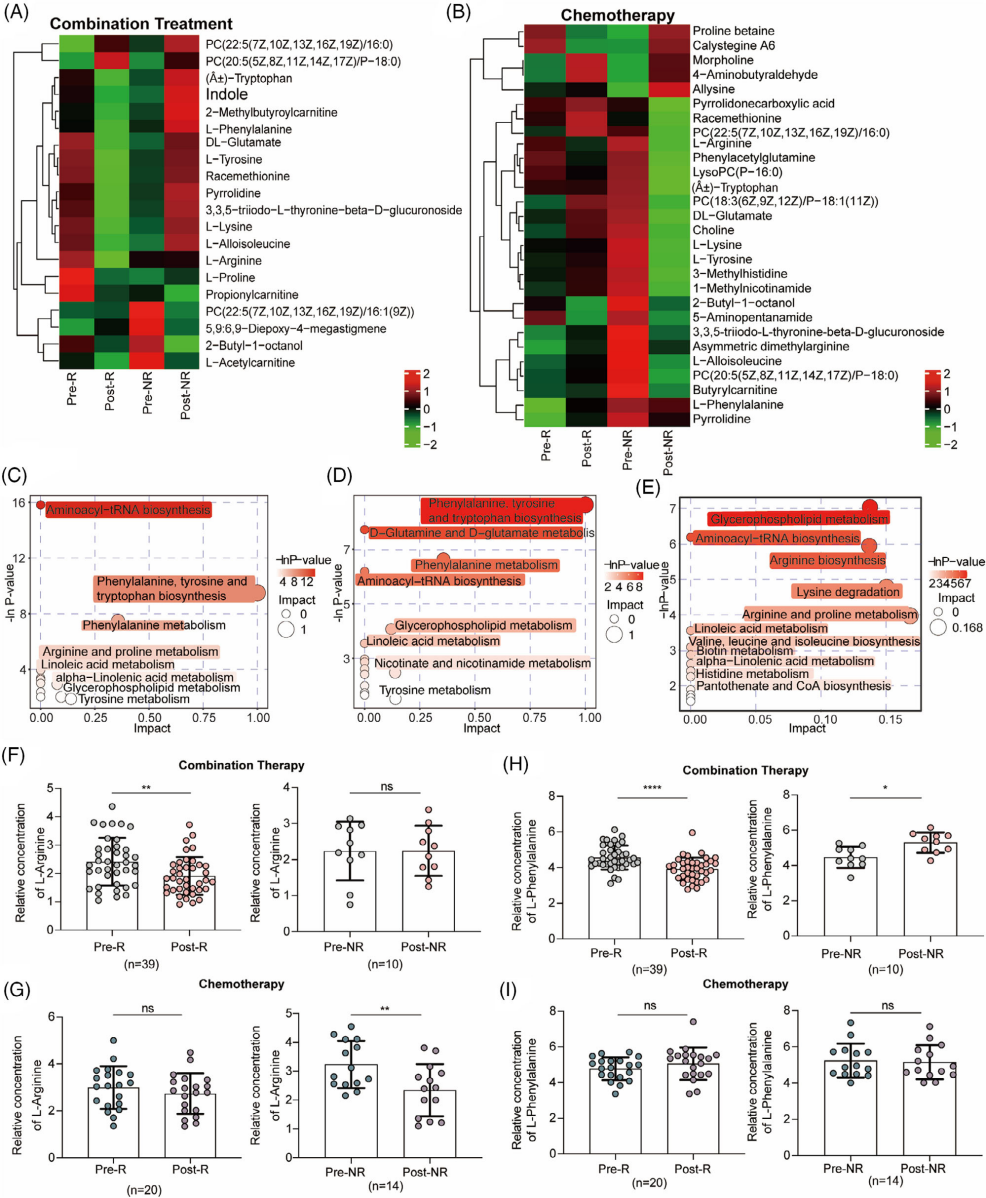
L‐phenylalanine concentrations were decreased in R patients and increased in NR patients. (A) Heatmap representing metabolite changes in patients before combination treatment compared with before. (B) Heatmap representing metabolite changes in patients after chemotherapy treatment compared with before. (C‒E) Bubble plot enrichment analysis of metabolic pathways of differential metabolites. (C) NR patients received combination treatment; (D) R patients received chemotherapy; (E) NR patients received chemotherapy. (F) The relative concentration of L‐arginine in responders (*n* = 39) and non‐responders (*n* = 10) before and after combination treatment. (G) The relative concentration of L‐arginine in responders (*n* = 20) and non‐responders (*n* = 14) before and after chemotherapy. (H) The relative concentration of L‐phenylalanine in responders (*n* = 39) and non‐responders (*n* = 10) before and after chemotherapy combination treatment. (I) The relative concentration of L‐phenylalanine in responders (*n* = 20) and non‐responders (*n* = 14) before and after chemotherapy.

We further focused on different metabolites that indicate the efficacy of combination treatment. First, we analyzed the different metabolites at baseline and after treatment in patients with NSCLC treated with combination treatment (Figure 3A) or chemotherapy alone (Figure 3B). A heatmap revealed differential metabolites, including amino acids and lipids (Figure 3A,B). Based on these differential metabolites, we analyzed the metabolic pathways in which the differential metabolites might be involved. In patients who benefitted from combination treatment, the metabolic pathways were mainly enriched in L‐phenylalanine and arginine metabolism (Figure 3C). Among the R group who received chemotherapy, the metabolic pathways were mainly enriched in L‐phenylalanine‐tyrosine metabolism (Figure 3D), whereas in NR group, they were mainly enriched in L‐arginine metabolism (Figure 3E). Furthermore, considering that L‐arginine and L‐phenylalanine metabolisms were the major enriched metabolic pathways, we compared the concentrations of L‐arginine and L‐phenylalanine. Compared to baseline, the concentration of L‐arginine decreased in patients who benefited from combination therapy (2.409 ± 0.8415 vs. 1.911 ± 0.6653; Figure 3F) and in NR patients receiving chemotherapy (3.233 ± 0.8189 vs. 2.343 ± 0.9018; Figure 3G). Additionally, the concentration of L‐phenylalanine showed opposite changes in patients after treatment with combination therapy (Figure 3H), which decreased after treatment in responders (4.559 ± 0.6762 vs. 3.919 ± 0.6351; left) and increased after treatment in non‐responders (4.463 ± 0.5965 vs. 5.299 ± 0.5755; right). However, there was no significant difference in the L‐phenylalanine concentration between baseline and post treatment in patients who received chemotherapy in both the R and NR groups (Figure 3I). Therefore, changes in the concentration of L‐phenylalanine may be an ideal indicator of the specificity of chemotherapy combined with anti‐PD‐1 antibody.

### 2.4 Changes in L‐phenylalanine concentration can reflect the efficacy of the combination

**FIGURE 4:**
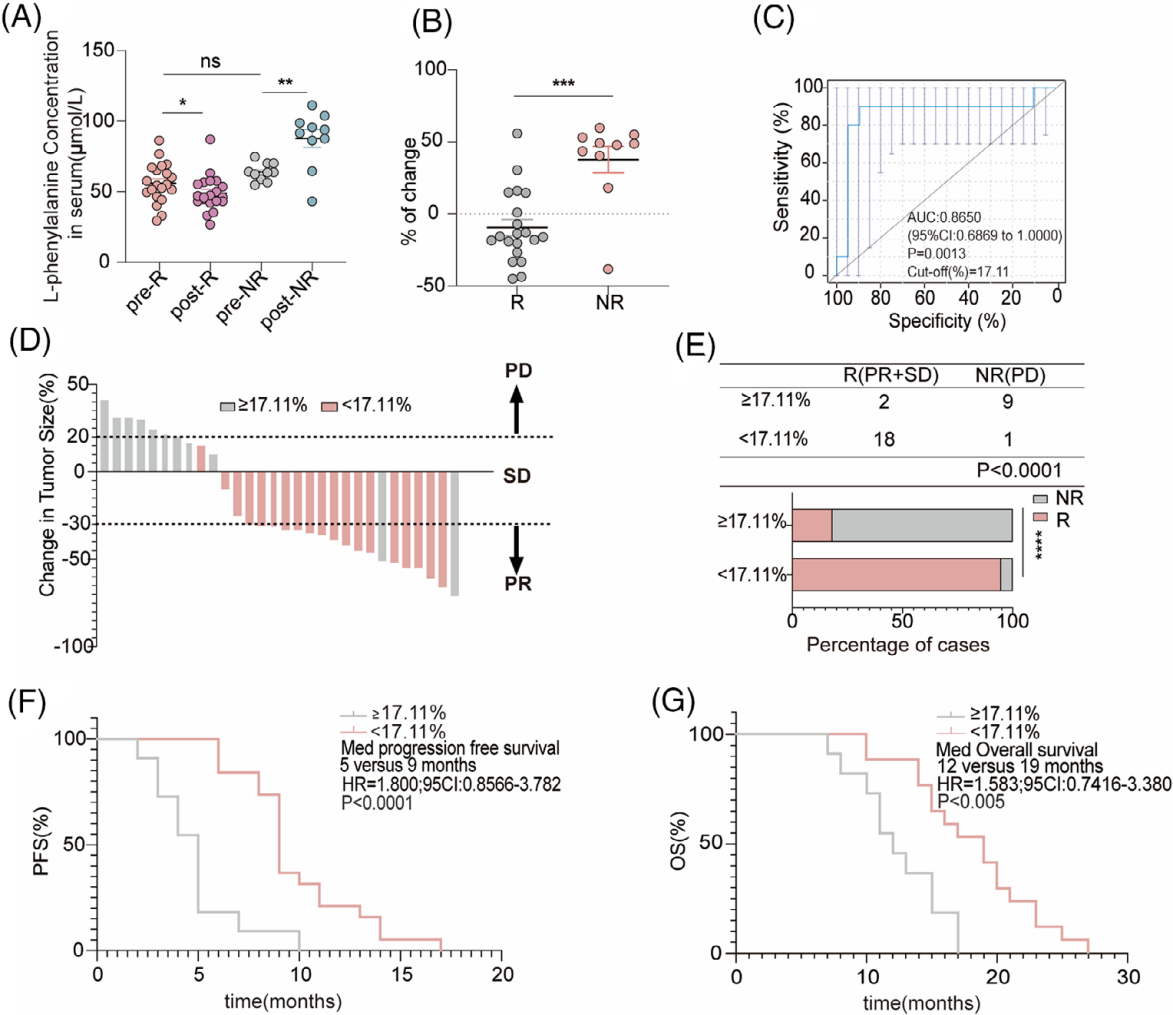
Changes in serum L‐phenylalanine levels reflect tumor response in NSCLC patients during chemotherapy combined with PD‐1Ab treatment. Patients treated with chemotherapy combined with the PD‐1Ab were assessed at baseline (BL), best response (R), and disease progression (PD). (A) L‐phenylalanine levels plotted at baseline (BL), at the best response moment (R) in responders (*n* = 20), and at the moment of disease progression (PD) in non‐responders (*n* = 10). (B) Percentage change in serum L‐phenylalanine in responders (*n* = 20) and non‐responders (*n* = 10) from baseline levels to four cycles after the start of treatment. A nonparametric Wilcoxon test was used to compare median L‐phenylalanine levels between responders and non‐responders. (C) ROC curve results distinguishing responders from non‐responders in the discovery set. (D) Bar chart showing the arrangement of the change values in the tumor volume change histogram. (E) Three‐line tables and statistical graphs showing the proportion of responders and non‐responders in each group higher or lower than the cutoff value. (F and G) Kaplan–Meier survival curves stratified by progression‐free survival (PFS) and overall survival (OS) according to patient changes.

Next, 20 R and 10 NR patients who received chemotherapy combined with an anti‐PD‐1 antibody used for non‐targeted metabolomics were selected to determine the concentration of L‐phenylalanine using ELISA. In line with the non‐targeted metabolomics results, the concentration of L‐phenylalanine in the serum of non‐responders increased after treatment; however, the concentration of L‐phenylalanine decreased in responders (Figure 4A). Based on the changing trend in L‐phenylalanine concentration at baseline and post treatment, we hypothesized that changes in serum L‐phenylalanine concentration might serve as a prognostic of the clinical benefit of combination treatment. Therefore, we designed a formula to visualize the changes by calculating the ratio of the L‐phenylalanine concentration ([post‐treatment concentration − baseline concentration] to the baseline concentration). As shown by the statistical results, the change values for the NR group were mostly positive, whereas those for the R group were mostly negative (Figure 4B). We evaluated the discriminatory power of L‐phenylalanine using receiver operating characteristic (ROC) curves. The area under the curve (AUC) for L‐phenylalanine was 0.8650. The best cutoff value for L‐phenylalanine changes indicated that the efficiency of combination treatment was 17.11% (Figure 4C). Subsequently, the patients were divided into two groups according to the cutoff value. Patients in the cutoff value ≥17.11% group showed progressive disease (PD) after combination treatment (Figure 4D). In the cutoff value ≥17.11% group, the objective response rate (ORR) was 18.18% (2/11), lower than in the cutoff value less than 17.11% group (ORR 94.74%, 18/19) (Figure 4E). Finally, we followed‐up on progression‐free survival (PFS) and (overall survival) OS. We found that patients in the cutoff value ≥17.11% group had shorter PFS (cutoff value ≥17.11% group: median PFS: 5 months; cutoff value <17.11% group: median PFS: 9 months) and OS (cutoff value ≥17.11% group: median OS: 12 months; cutoff value <17.11% group: median OS: 19 months) compared to patients in the cutoff value less than 17.11% group (Figure 4F,G). Therefore, we identified and validated changes in L‐phenylalanine concentration as a prognostic of response to combination therapy.

### 2.5 Changes in L‐phenylalanine concentration preceded radiographic size changes

**FIGURE 5:**
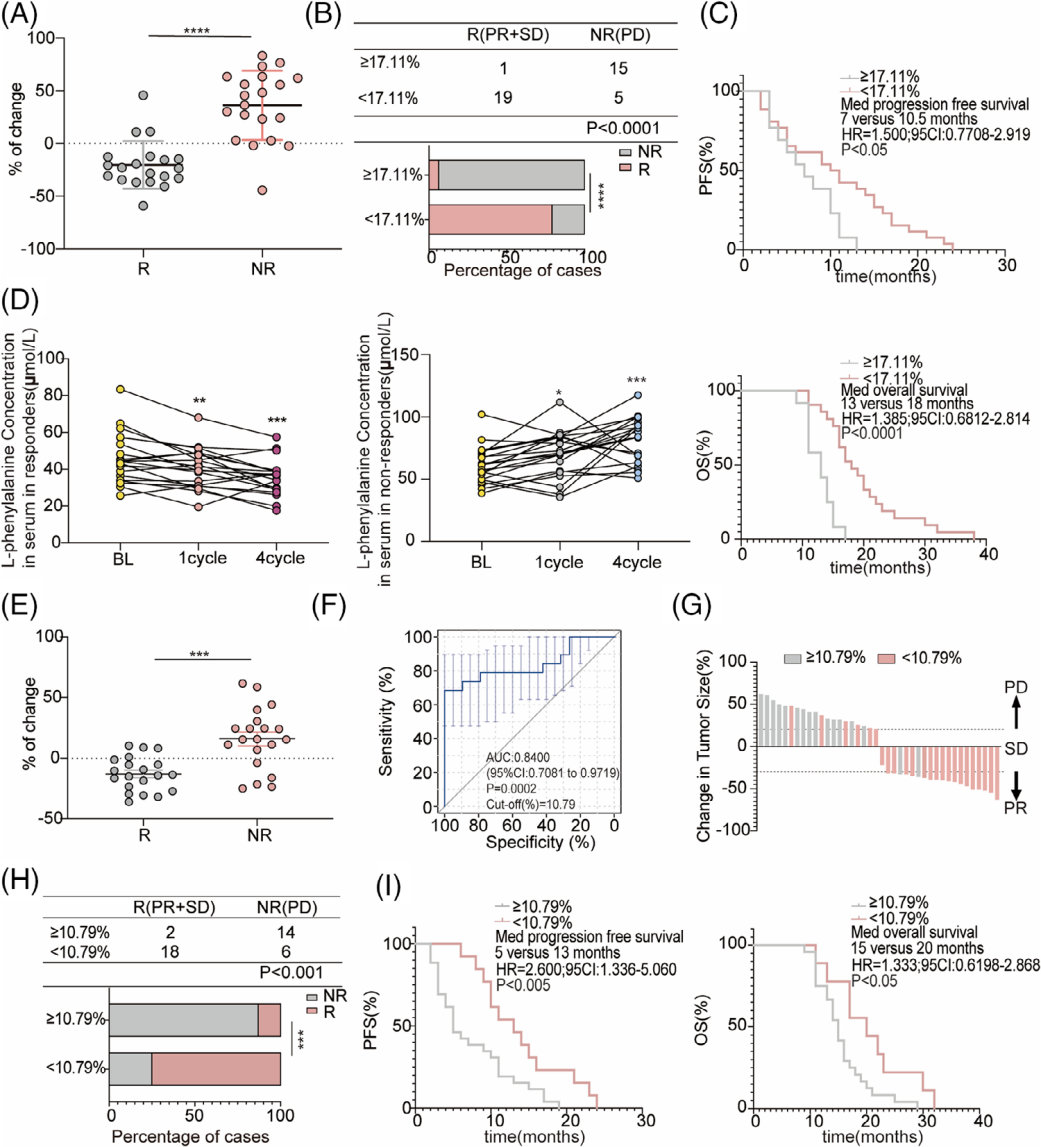
Fold‐change of L‐phenylalanine relative concentration is correlated with the PD‐1Ab efficacy in cancer patients. (A) Percentages of change in serum L‐phenylalanine in responders (*n* = 20) and non‐responders (*n* = 20) from baseline levels to four cycles after the start of treatment in validation 1. (B) Three‐line tables and statistical graph show the proportion of responders and non‐responders in each group higher or lower than the cutoff value of the discovery cohort. (C) Kaplan–Meier survival curves stratified progression‐free survival (PFS, up) and overall survival (OS, down) by serum L‐phenylalanine change in patients. (D) Results of L‐phenylalanine levels are plotted in baseline (BL), and at the best response moment (responders [*n* = 20], left) or the progression disease moment (non‐responders [*n* = 20], right) in validation 1. (E) Percentages of change in serum L‐phenylalanine in responders (*n* = 20) and non‐responders (*n* = 20) from baseline levels to one cycle after the start of treatment in validation 1. (F) ROC curves result in distinguishing responders from non‐responders for the discovery set. (G) Bar chart showing the arrangement of the change values in the tumor volume change histogram according to R. (H) Three‐line tables and statistical graph show the proportion of responders and non‐responders in each group higher or lower than the cutoff value. (I) Kaplan–Meier survival curves stratified PFS (left) and OS (right) by change in patients.

Forty patients who received chemotherapy combined with anti‐PD‐1 antibody therapy (R: *n* = 20; NR: *n* = 20) were randomly selected for the validation cohort. Changes in L‐phenylalanine concentration in the NR group were significantly higher than in the R group (Figure 5A). Similarly, the samples were divided into two groups using a cutoff value of 17.11%. Consistent with previous results in the discovery cohort, the ORR in the cutoff value ≥17.11% group (6.25%, 1/16) was lower than in the less than 17.11% group (79.17%, 19/24) (Figure 5B). PFS and OS in the cutoff value ≥17.11% group were shorter than in the cutoff value less than 17.11% group (Figure 5C).

However, although the changes in serum L‐phenylalanine at four cycles after therapy reflected the clinical efficacy of combination therapy, clinicians tend to prefer using CT to evaluate the therapeutic effect after at least four cycles of treatment. We further investigated whether the change in serum L‐phenylalanine concentration after one cycle of therapy was a good prognostic of clinical efficacy. Therefore, we dynamically analyzed the changes in serum L‐phenylalanine concentrations in patients in the validation cohort at different time points after receiving combination treatment. The L‐phenylalanine concentration decreased in responders one cycle after treatment, whereas the concentration of L‐phenylalanine in non‐responders increased one cycle after treatment (Figure 5D). Furthermore, we undertook an extended follow‐up of 20 participants who were given combination therapy, and their serum L‐phenylalanine concentrations were measured upon disease progression. At the onset of disease progression, all patients demonstrated an individual variation in the elevation levels of L‐phenylalanine (Figure S2A). Moreover, the L‐phenylalanine concentrations from two arbitrarily selected responders were monitored at baseline and during each subsequent cycle. The findings suggested a slight increase or decrease in L‐phenylalanine concentration when patients exhibited either stable disease or partial response (Figure S2B,C).

Next, we used the same formula to visualize the changes in L‐phenylalanine concentration one cycle after treatment and found that the change values for the NR group were mostly positive, whereas those for the R group were mostly negative (Figure 5E), consistent with the results in Figure 4B. The AUC was 0.8400, and the best cutoff value was 10.79% (Figure 5F). Patients in the validation cohort were divided into two groups according to the cutoff value in Figure 5F. Most patients in the cutoff value ≥10.79% group achieved PD after combination treatment (Figure 5G). In the cutoff value ≥10.79% group, ORR was 12.5% (2/16), lower than in the cutoff value less than 10.79% group (ORR 75%, 18/24) (Figure 5H). Patients with the cutoff value less than 10.79% had significantly better PFS and OS than patients with the cutoff value ≥10.79% (Figure 5I). Taken together, changes in L‐phenylalanine concentration can be used as an early clinical prognostic diagnostic indicator before CT evolution.

### 2.6 Prospective validation of the discriminant model

**FIGURE 6:**
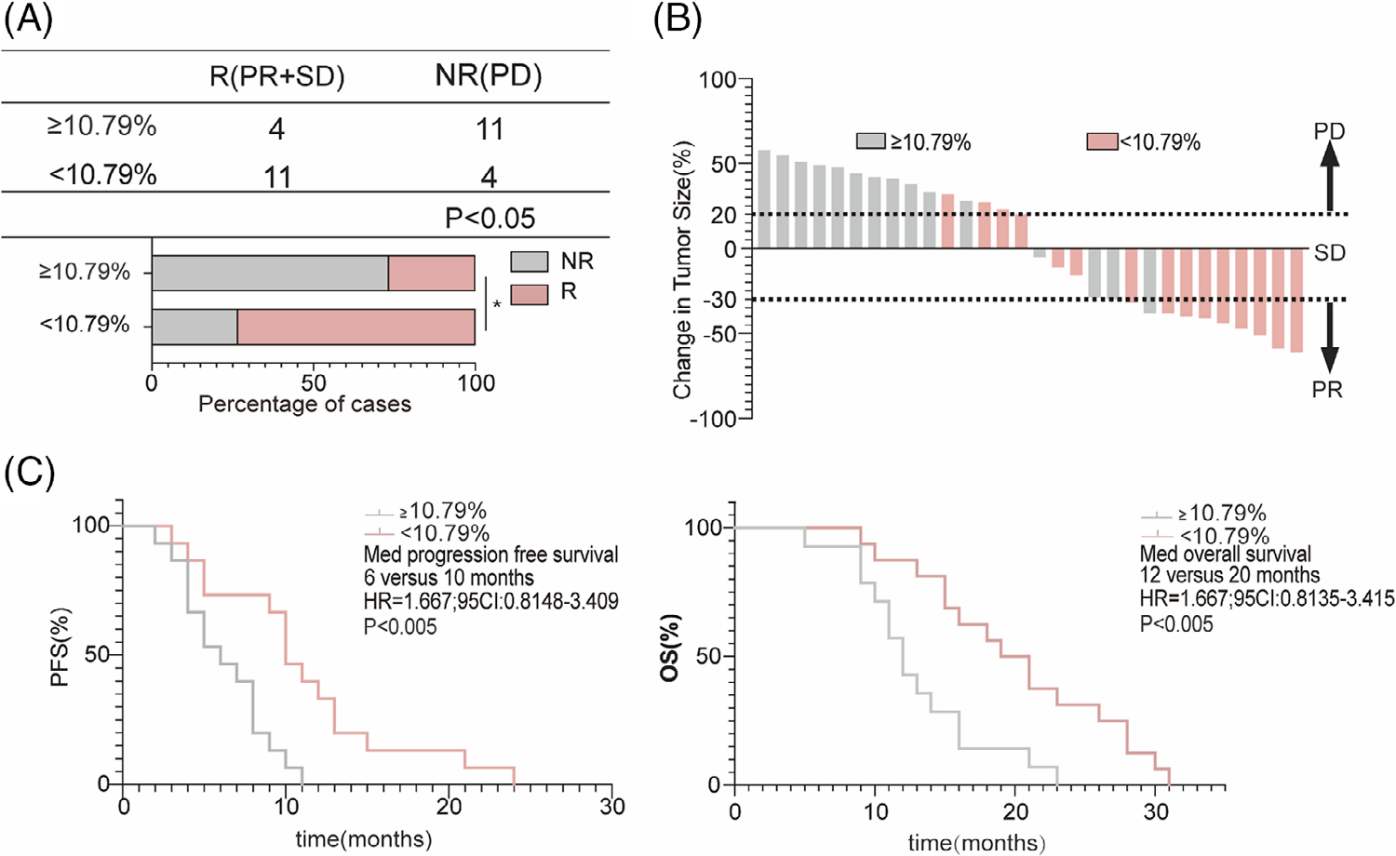
Fold‐change of L‐phenylalanine reflects that treatment of chemotherapy combined with PD‐1Ab efficacy in validation 2. (A) Three‐line tables and a statistical graph showing the proportion of responders (*n* = 15) and non‐responders (*n* = 15) in each group higher or lower than the cutoff value. (B) The tumor sizes provided under the images were evaluated by physicians. (C) Kaplan–Meier survival curves stratified progression‐free survival (PFS, left) and overall survival (OS, right) by change in patients.

Finally, to verify the early clinical predictive efficiency of L‐phenylalanine levels, we conducted a prospective cohort study (R: *n* = 15; NR: *n* = 15). The serum L‐phenylalanine concentration of each patient was measured at baseline and one cycle after the treatment cycle. Patients in the prospective cohort were divided into two groups based on a cutoff value of 10.79% in Figure 5F. ORR in the cutoff value ≥10.79% group (26.67%, 4/15) was lower than in the cutoff value less than 10.79% group (73.33%, 11/15) (Figure 6A). CT images of the patients were obtained after four cycles of treatment; tumors of most patients in the cutoff value less than 10.79% group shrank after therapy, while tumors in the cutoff value ≥10.79% group showed continuous progression (Figure 6B). The median PFS of patients with the cutoff value ≥10.79% was 10 months (95% confidence interval [CI]: 0.8148–3.409), significantly shorter than in patients with the cutoff value less than 10.79% (Figure 6C). Meanwhile, the median OS in the cutoff value less than 10.79% group was significantly longer than in the cutoff value ≥10.79% group (Figure 6C).

## 3 DISCUSSION

This study identified a prognostic model based on changes in L‐phenylalanine concentration between baseline and after four cycles of treatment, based on the results of non‐targeted metabolomics. The model was validated by collecting patient imaging results and statistically analyzing the PFS and OS. Retrospective validation sets were collected to validate the prognostic models identified in the discovery sets. To achieve early prediction of PD‐1Ab combination therapy and chemotherapy, we established an earlier prognostic model based on the changes in L‐phenylalanine concentration between one cycle after treatment and baseline in the retrospective validation set and collected specimens as prospective validation sets. This prognostic model can be easily used by doctors to select patients who may benefit from a combination of chemotherapy and PD‐1Ab treatment, thus providing a promising individualized strategy for this widely used combination therapy.

L‐phenylalanine, an essential amino acid,^19^ has a stable metabolic pathway and concentration fluctuation,^20^ with a physiological concentration in the body ranging from 35 to 120 µmol/L.^21^ In our study, the concentration of phenylalanine was within the normal range before and after the combined treatment. Additionally, the patient cohort in the current study was small, and the model should be confirmed in a large cohort with multicenter controls.

In our clinical cohort and metabolomic analysis, we used the change in L‐phenylalanine concentration as the basis for the predictive model, in part because its concentration only changed before and after treatment with PD‐1Ab combined with chemotherapy, whereas the change before and after chemotherapy alone was not significant. According to the literature, the increase in the concentration of L‐phenylalanine in the blood has been reported in diseases such as HIV‐1 infection, bone health, sepsis, burns, and malignant tumors.^22^, ^23^, ^24^, ^25^, ^26^, ^27^ The changes in the concentration and roles of different types of tumors were not the same. Our model used only NSCLC patients treated with PD‐1Ab combined with chemotherapy. Metabolic biomarkers should be further evaluated in patients receiving combination therapy using serum metabolic panels to predict the clinical effects of combination immunotherapy in patients with all tumor types. Some studies have reported that increasing the concentration of certain amino acids such as arginine,^28^ taurine,^29^ glutamine,^30^ and others can improve the therapeutic effect of ICB and the antitumor function of T cells. Our study found that after combination therapy, the L‐phenylalanine levels of non‐responders slightly increased, whereas those of responders significantly decreased. This change in concentration was used as a prognostic indicator before the tumor imaging changed during the treatment process. In addition to identifying this metabolic biomarker, the study also provides new insights into improving the effectiveness of combination therapy. We propose a treatment strategy that combines PD‐1 blockade and intervention to improve clinical efficacy.

L‐phenylalanine is ingested into cells, and hydrogen peroxide (H_2_O_2_) and phenyl ketone acids are produced through the oxidative deamination of L‐phenylalanine, which inhibits T‐cell proliferation and signaling pathway damage.^31^, ^32^ This may partially explain why serum L‐phenylalanine levels in the non‐benefit group of patients receiving combined immunotherapy increased further.

To our knowledge, this is the first large cohort study to use metabolic biomarkers to indicate the clinical response of NSCLC patients to chemotherapy combined with immunotherapy. The discrimination model established in this study provides a feasible and convenient strategy for the personalized application of chemotherapy combined with immunotherapy. The high accuracy of the model established in our study in identifying the possibility of a therapeutic response ensures further development of prospective clinical trials.

## 4 CONCLUSIONS

Our study demonstrated a serum metabolite prognostic model for changes in L‐phenylalanine concentrations after chemotherapy combined with PD‐1 treatment in patients with NSCLC to assess the response. The model was validated through retrospective and prospective analyses, demonstrating its potential as an early indicator of therapeutic outcomes.

## 5 MATERIALS AND METHODS

### 5.1 Study population

The clinical data of patients diagnosed with NSCLC by postoperative/biopsy pathology from February 2019 to June 2023 were collected at the First Affiliated Hospital of Zhengzhou University. The clinical data collection and specimen collection processes for this project were approved by the Zhengzhou University Life Science Ethics Review Committee. All patients provided written informed consent [approval number: 2021‐KY‐1105‐002].

Inclusion criteria: patients aged 18–65 years, not receiving PD‐1 monoclonal antibody and other immunotherapy before enrollment, no sensitive gene mutations, ECOG score ≤2 points, all tumor tissue specimens confirmed by pathological diagnosis as NSCLC. The chemotherapeutic regimen consisted of pemetrexed plus platinum. The combination therapy regimen comprised antibodies against PD‐1, pemetrexed + platinum. Lung CT tomography was performed before starting the treatment plan. Patients with an unclear pathological diagnosis, those lost to follow‐up, and those who withdrew from the PD‐1 monoclonal antibody treatment owing to adverse reactions or side effects were excluded.

### 5.2 Clinical data and follow‐up

Clinical data include age, gender (female vs. male), clinical stage (stage 1–2 vs. stage 3–4), ECOG score, PD‐L1 expression (CPS standard), surgical history (yes/no), number of pre‐treatment metastases, whether distant metastasis (brain, pleura, bone) occurred, previous treatment regimen, and line of treatment.

All patients underwent lung CT scans every 2 months after treatment initiation. For patients with lymph node metastasis, whole‐body PET/CT was performed every 6 months to determine the presence of distant metastases. Furthermore, postoperative follow‐up included abdominal ultrasonography, tumor markers, routine blood tests, and blood biochemical tests. PFS is defined as the period from the start of treatment to the time when the sum of tumor diameters increases by at least 20% of its volume or when new metastatic lesions appear. OS was defined as the time from the beginning of treatment to the last follow‐up or death.

### 5.3 Non‐targeted metabolomics analysis

The patient's peripheral blood was obtained and centrifuged at 400 × *g* for 10 min within 1 h. The upper serum was collected, and stored in a −80°C refrigerator. The relative abundance of serum metabolites was determined using liquid chromatography‐mass spectrometry (LC‐MS). We performed a comprehensive and systematic identification and analysis of endogenous metabolites in the serum and carried out cluster analysis based on the qualitative analysis results to identify the key metabolites that differed between the groups.

### 5.4 Elisa assay

L‐phenylalanine levels were measured in serum samples. The concentration of L‐phenylalanine was assessed using an ELISA kit (Abcam, ab241000) following the manufacturer's instructions. In this assay, L‐phenylalanine is metabolized by the simultaneous formation of NADH, which reacts with a probe to generate an absorbance that can be monitored colorimetrically at 450 nm. The L‐phenylalanine concentration was calculated according to the standard curve obtained using the kit.

### 5.5 Statistical analysis

We used the chi‐square test to compare categorical variables such as age and sex; the results are presented as absolute counts and three‐line graphs. For continuous variables in the non‐targeted metabolomics results, we used the orthogonal partial least squares discriminant analysis (OPLS‐DA) method to reduce dimensionality and screen important variables. First, the categorical variables were used for univariate logistic regression analysis, and all factors with *p*‐values less than 0.05 were included in the multivariable stepwise analysis. We needed to identify the factors that can affect patient outcomes and develop models for these factors. To evaluate the accuracy and sensitivity of the predictive model, a retrospective validation was set 1. Subsequently, the predictive performance of the model was compared with that of the radiological examination to evaluate the applicability of the clinical model. In prospective validation set 2, we used the highest threshold of Youden's index (the sum of specificity and sensitivity) to divide the patients into high‐ and low‐risk groups. Finally, the PFS and OS of the two patient groups were verified using Kaplan–Meier (KM) curves. All analyses were performed using Prism 8.0, SPSS, and R software (survival and pROC).

## AUTHOR CONTRIBUTIONS

**Y.Z**. and **L.W**. designed the experiments. **Y.L., Y.P**., and **L.Z**. performed the experiments. **Q.Z., Y.H., C.L., J.S**., and **Y.Q**. helped with the experiments. **Y.L., Y.P., L.Z**., and **Q.Z**. prepared the manuscript. All authors have read and approved the final version of the manuscript.

## CONFLICT OF INTEREST STATEMENT

The authors declare no conflicts of interest.

## ETHICS STATEMENT

The clinical data and specimen collection processes for this project were approved by the Zhengzhou University Life Science Ethics Review Committee (approval number 2021‐KY‐1105‐002). For studying patients, an informed consent was obtained from all participants, and written consent was obtained from the study participants.

## Supporting information



Supporting Information

## Data Availability

Please contact the corresponding author for data requests. Untargeted metabolomics data that support the findings of this study will be available in Metabolights at https://www.ebi.ac.uk/metabolights (MTBLS code:11232).
