# Supplementary material for: Changes in L‐phenylalanine concentration reflect and predict response to anti‐PD‐1 treatment combined with chemotherapy in patients with non‐small cell lung cancer
Source: MedComm (2020). 2025 Feb 17;6(3):e70100. doi: 10.1002/mco2.70100 (PMC11832432; doi:10.1002/mco2.70100)
Supplement: Supplementary file 1 — Supporting Information [file MCO2-6-e70100-s001.docx]

**Changes in L-phenylalanine concentration reflect and predict response to anti-PD-1 treatment combined with chemotherapy in patients with non-small cell lung cancer**

Yaqing Liu^1,2#^, Yu Ping^1#^, Liubo Zhang^1#^, Qitai Zhao^1^, Yachang Huo^1^, Congcong Li^1^, Jiqi Shan^1^, Yanwen Qi^2^, Liping Wang^2*^, Yi Zhang^1,3,4,5,6*^

^1^Biotherapy Center and Cancer Center, the First Affiliated Hospital of Zhengzhou University, Zhengzhou, Henan, China

^2^Department of Oncology, the First Affiliated Hospital of Zhengzhou University, Zhengzhou, Henan, China

^3^State Key Laboratory of Esophageal Cancer Prevention and Treatment, Zhengzhou, Henan, China

^4^School of Life Sciences, Zhengzhou University, Zhengzhou, Henan, China

^5^Tianjian Laboratory of Advanced Biomedical Sciences, Academy of Medical Sciences, Zhengzhou University, Zhengzhou, Henan, China

^6^School of Public Health, Zhengzhou University, Zhengzhou, Henan, China

*Corresponding author:

Yi Zhang, the First Affiliated Hospital of Zhengzhou University, No. 1 Jianshe Road, Zhengzhou, Henan 450052, China. Phone: 8637-1662-95320; Fax: 8637-1669-70906; E-mail: yizhang@zzu.edu.cn;

Liping Wang, the First Affiliated Hospital of Zhengzhou University, No. 1 Jianshe Road, Zhengzhou, Henan 450052, China. E-mail: wlp@zzu.edu.cn.


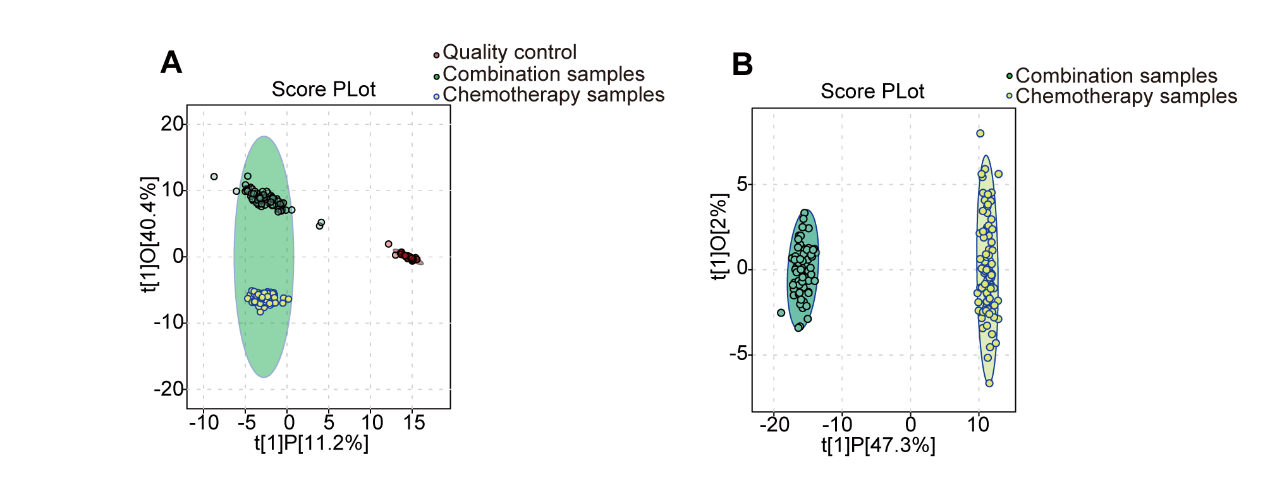


Figure S1. Examination of the metabolic profiles of serum specimens.


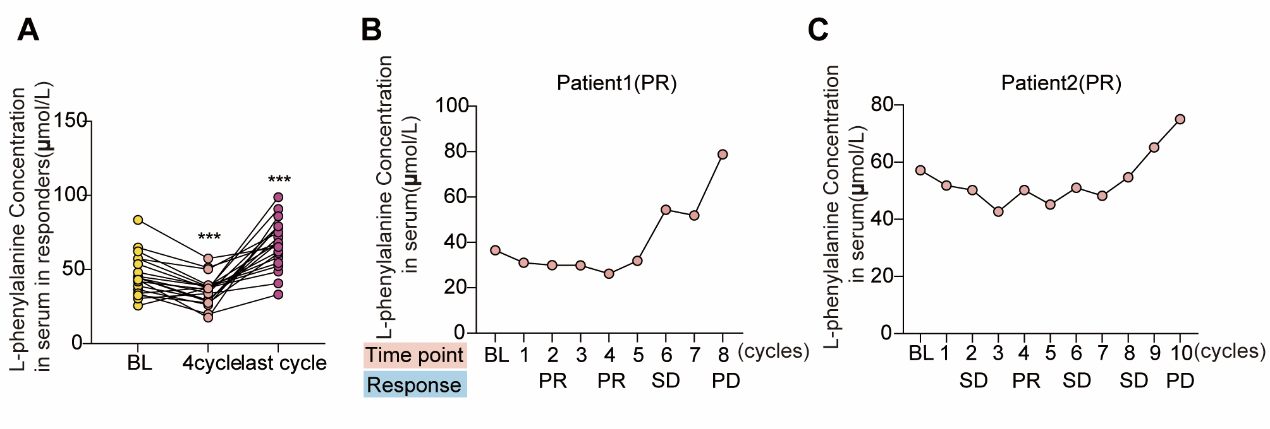


Figure S2. The increase in L-phenylalanine concentration indicates disease progression in NSCLC patients.
